# Pediatric shoulder instability: epidemiology, etiology, diagnosis and treatment

**DOI:** 10.1007/s00402-026-06246-y

**Published:** 2026-03-06

**Authors:** Alp Paksoy, Philipp Moroder, Doruk Akgün

**Affiliations:** 1 Charité University Hospital, Berlin, Germany; 2 Schulthess-Klinik, Zurich, Switzerland

**Keywords:** Pediatric shoulder instability, Arthroscopic stabilization, Sports injuries in youth, Anterior shoulder instability, Posterior shoulder instability, Multidirectional instability

## Abstract

**Abstract:**

Shoulder instability is increasingly prevalent among pediatric and adolescent populations due to growing participation in competitive sports at younger ages. However, the literature remains challenging to apply clinically, as it often fails to distinguish between different developmental stages, leading to potential overtreatment or undertreatment. This review aims to categorize types of shoulder instability in young patients, propose a diagnostic approach, and summarize current management strategies based on available evidence. Shoulder dislocations are rare in skeletally immature patients, with the highest risk observed in those aged 14–18 years. Younger children, particularly those under ten, are less prone to dislocations due to the relative strength of their ligaments compared to bone. Diagnosis relies on history, physical examination, and imaging modalities such as radiographs, computed tomography (CT), and magnetic resonance imaging (MRI). Special attention is required for functional posterior instability, which is frequently misdiagnosed. Treatment decisions—whether conservative or surgical—remain controversial. Conservative management, including immobilization and rehabilitation, is the first-line approach for primary anterior dislocations, particularly in children under 12. However, adolescents aged 12–16 face a high risk of recurrence, making early surgical stabilization a viable option. Arthroscopic stabilization is the preferred surgical technique, especially for athletes. In cases of recurrent instability with significant glenoid bone loss, the Latarjet procedure or iliac crest bone grafting may be indicated. Posterior instability, though rare, follows treatment principles similar to those in adults, with a primary emphasis on rehabilitation. Functional posterior instability responds well to neuromuscular electrical stimulation. Multidirectional instability, often associated with ligamentous laxity, is primarily managed nonoperatively, but surgical stabilization may be necessary if symptoms persist. In conclusion, pediatric shoulder instability is complex and requires an individualized approach. Understanding age-specific anatomical and physiological differences is crucial for optimizing treatment outcomes and preventing long-term complications.

**Level of evidence:**

Level 5.

## Introduction

Shoulder instability is increasingly common in the paediatric and adolescent population [17], as participation in competitive sports at younger ages continue to increase in number and intensity [3; 40]. The literature on pediatric shoulder instability is challenging to apply in clinical practice because most of the studies do not distinguish between children under 10 years old, adolescents with open growth plates, and those with closed growth plates [29]. Moreover, since the physiology of pediatric patients changes with age, variations in the age ranges included across studies make comparisons difficult [53]. Without clear epidemiological data, many of these patients are likely being treated based on generalizations from adult data, which may result in overtreatment or undertreatment. This review provides information on the various types of shoulder instability common in young patients, outlines a basic diagnostic approach, and reviews the literature guiding management principles in this specific population. For the purposes of this review, the terms “children” and “paediatric” population will refer to patients aged between five and 14 years, whose glenohumeral growth plates are still open.

## Epidemiology

Shoulder dislocations are relatively rare in skeletally immature patients. Pediatric population under the age of 20 are at most significant risk of developing a shoulder dislocation, while the same pathology is rare in children younger than ten years [33, 34, 66]. Only 2% of all traumatic glenohumeral dislocations occur in patients aged between 1 and 10 years [4, 55]. According to the systematic review of the literature prior to 2015 conducted by Olds et al., 14-18-year-old patients are 24 times more likely to experience redislocation than those aged 13 years and below, and those with a closed physis are 14 times more likely to dislocate than those with an open physis [48, 53]. This also explains the lack of data for optimal standard treatment of pediatric patients with shoulder instability. In the young population with an immature skeleton, the physes remain open, and shoulder injuries are more likely to result in physeal or metaphyseal fractures rather than ligamentous ruptures or dislocations [36, 38], since ligaments in young children are seven times stronger than the bone [28].

## History and physical examination

The diagnosis of shoulder instability can often be made based on a thorough history to assess for pain, mechanism of injury, prior instability events, and other associated symptoms. Physical examination includes common provocative tests for the assessment of anterior instability such as the anterior load and shift test, the anterior apprehension test, and the relocation sign [19, 24, 56]. A positive Jerk test, Kim test, or push–pull test may suggest posterior instability [49]. Specific instability tests such as the Beighton score, and the Sulcus and Gagey signs should be used to analyze capsular and general ligamentous hyperlaxity and hypermobility, which have been associated with glenohumeral joint instability [2, 8, 14, 23, 57]. Moreover, a careful history is important to assess for family history of connective tissue disorders such as Ehlers Danlos syndrome for the patient with recurrent shoulder instability [25]. The clinical examination should be completed with sensory and motor examination for neurological involvement and by evaluating muscular imbalances especially scapulothoracic motion, rotator cuff strength, and periscapular and core stabilizing muscles [53]. Traumatic shoulder instability may be secondary to an epileptic seizure, which is why it is preferable to refer the patients to the pediatric neurology unit to perform a precise assessment [29].

In the pediatric population, patients with functional posterior instability need close attention, as they are often misdiagnosed or not diagnosed at all. Type B1 instability according to Moroder et al. due to pathological activation pattern of the rotator cuff as well as periscapular muscles has been identified to affect up to 3% of a young and active population with mostly atraumatic onset of first symptoms under the age of 16 years. It can develop over a period of time as an accumulative result of repetitive microtraumatic load and not clearly caused by an initial traumatic event [29, 49].

## Imaging

Radiographic workup includes true anterior-posterior, axillary, and scapula Y view of the affected shoulder. Standard radiographs are important to identify an acute or chronic locked dislocation, fractures or assess acromion morphology and open physes. In patients with open physes, radiographs should be performed prior to reduction to assess for a proximal humeral physeal fracture [42].

Computed tomography (CT) imaging provides precise assessment of fractures, humeral and glenoid defects, offering three-dimensional (3D) reconstruction of the bony anatomy. However, its use in the paediatric population should be balanced against the potential downstream effects of radiation.

**Figure 1:**
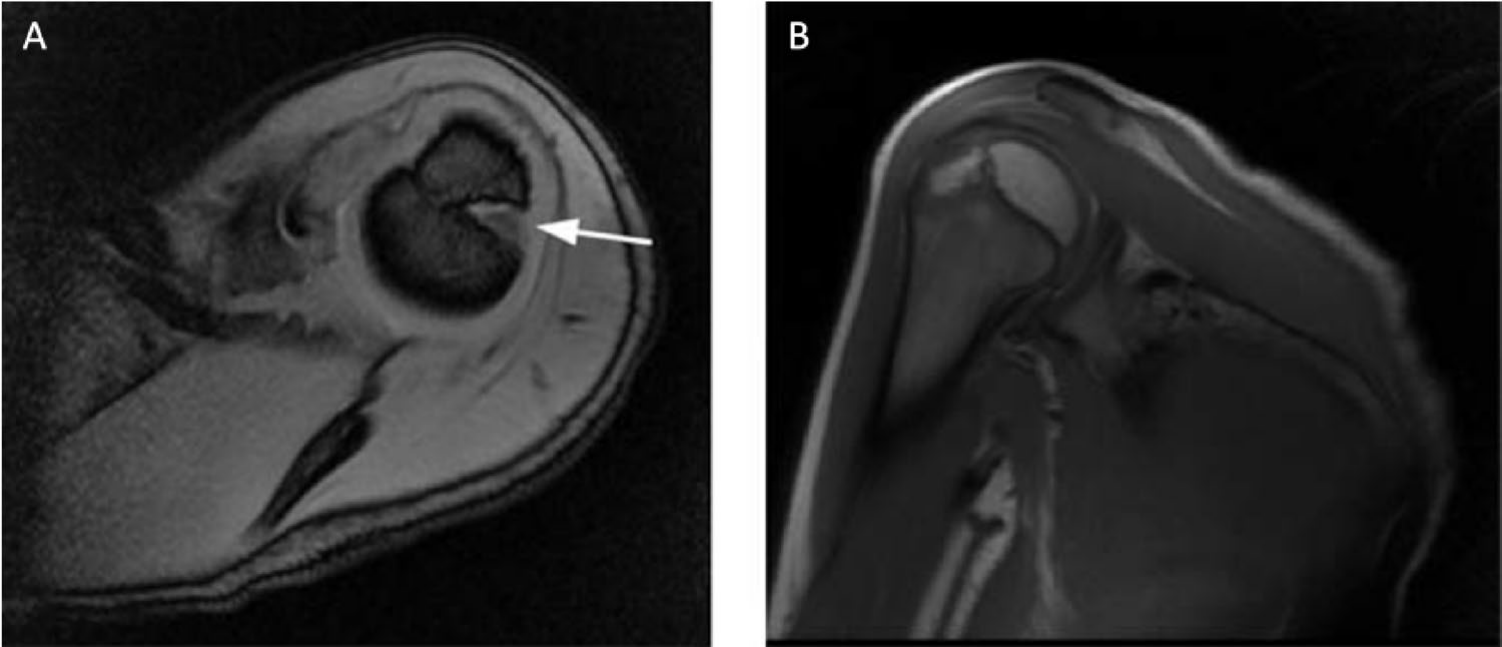
Axial (A) and coronal (B) T1-weighted MRI images of the shoulder in a 9-year-old patient. The images were obtained one week after reduction of an anterior shoulder dislocation was performed in the emergency department. The axial view demonstrates a Hill-Sachs lesion at the proximal humeral physis (arrow). This lesion is not visible on the coronal view. Figure taken from Li et al. 2013 [36]. Reprinted with permission from Wolters Kluwer Health, Inc [36]

**Figure 2:**
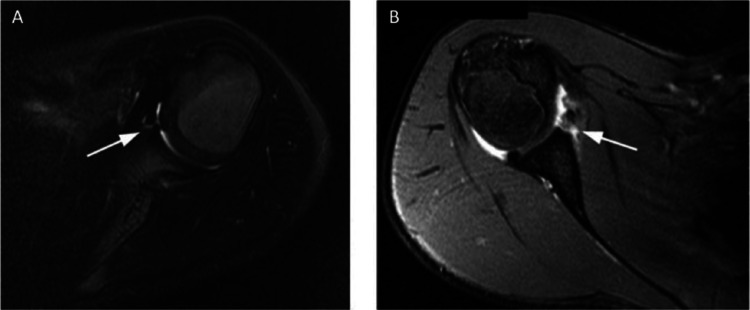
(A) Postreduction axial T2-weighted MRI of the shoulder in a 9-year-old boy who had an anterior glenohumeral dislocation. The anteroinferior capsulolabral tissue (arrow) was intact, with no evidence of a Bankart lesion in all views. (B) Postreduction axial T2-weighted MRI of the shoulder demonstrating a bony Bankart lesion (arrow) in a 14-year-old boy with an anterior glenohumeral dislocation. Figure taken from Li et al. 2013 [36]. Reprinted with permission from Wolters Kluwer Health, Inc [36]

Magnetic resonance imaging (MRI) is important for assessing the extent of injuries to the joint capsule, glenohumeral ligaments, labrum, and cartilage. MRI is recommended after first time dislocations to differentiate between various possible etiologies (Figs. 1 and 2). In comparison to CT scan, the lack of radiation wit MRI may be of more value in the paediatric population. When assessing MRIs in the paediatric population, the location and presence of ossification centres and physes should be carefully considered; as these can be mistaken for glenoid bone injuries, particularly the anterior glenoid ossification centre [53].

## Treatment

There are only few studies focused specifically on shoulder instability in pediatric patients, particularly those who are skeletally immature. This is why the decision between conservative and operative treatment for shoulder instability, as well as the appropriate timing for surgery, remains a topic of discussion [7, 16, 20, 22, 39, 41]. Traditionally, conservative treatment has been considered the gold standard for a primary shoulder dislocation in young patients but recent evidence suggests that early surgical intervention can reduce the incidence of subsequent episodes of glenohumeral instability [16, 20, 45, 61, 64].

### Anterior instability

#### First episode

The appropriate first-line management of a primary anterior shoulder dislocation has been conservative treatment consisting of a period of sling immobilization following by appropriate physical therapy and rehabilitation. Good functional outcomes and fast recovery after conservative treatment in children have been reported [10; 28], with recurrence rates ranging between 6% and 33% [10, 52, 58]. However, it is possible that these rates may reflect unrecognized morbidity after nonoperative management, such as dissatisfaction or failure to return to sport (e.g., ongoing apprehension), ongoing instability events not requiring physician reduction (self/spontaneous reductions or subluxation events), or a bias among surgeons toward the early stabilization of patients whom they perceive to be high risk. Moreover, recurrence rates vary significantly with age, with higher frequencies seen in adolescents. Hovelius et al. analyzed in a prospective multicenter study, patients who were initially managed nonoperatively after a primary anterior shoulder dislocation and showed that the youngest group (12–16 years) had the highest rate of recurrent instability followed by those aged 17–19 [22]. On the other hand, other studies have found that children aged 13 years and less are less likely to experience recurrent instability than those aged 14–18 [48, 51]. Several studies have analyzed the duration of immobilization in peadiatric population but none have found a relationship with the potential for recurrence [21, 31, 36]. Furthermore, physical therapy may be impractical or of limited benefit in very young patients (e.g. under 10 years old) [36].

Even with appropriate nonsurgical treatment, patients aged 12–16 years have considerably increased risk of developing recurrent instability after a first-time dislocation episode. Therefore, the role of early operative stabilization should be well understood in this paediatric population. A primary traumatic shoulder dislocation in pre-pubertal patients should be considered a distinct pathoanatomical entity; greater elasticity of the articular capsule and the glenoid labrum of these patients are less inclined to experience injuries after trauma. Moreover, the articular capsule inserts near to the border of the glenoid and might have a greater tension, preventing redislocations. So, they are less inclined to have detachment of the anterior labrum (Bankart lesion) [11, 52, 54].

**Figure 3:**
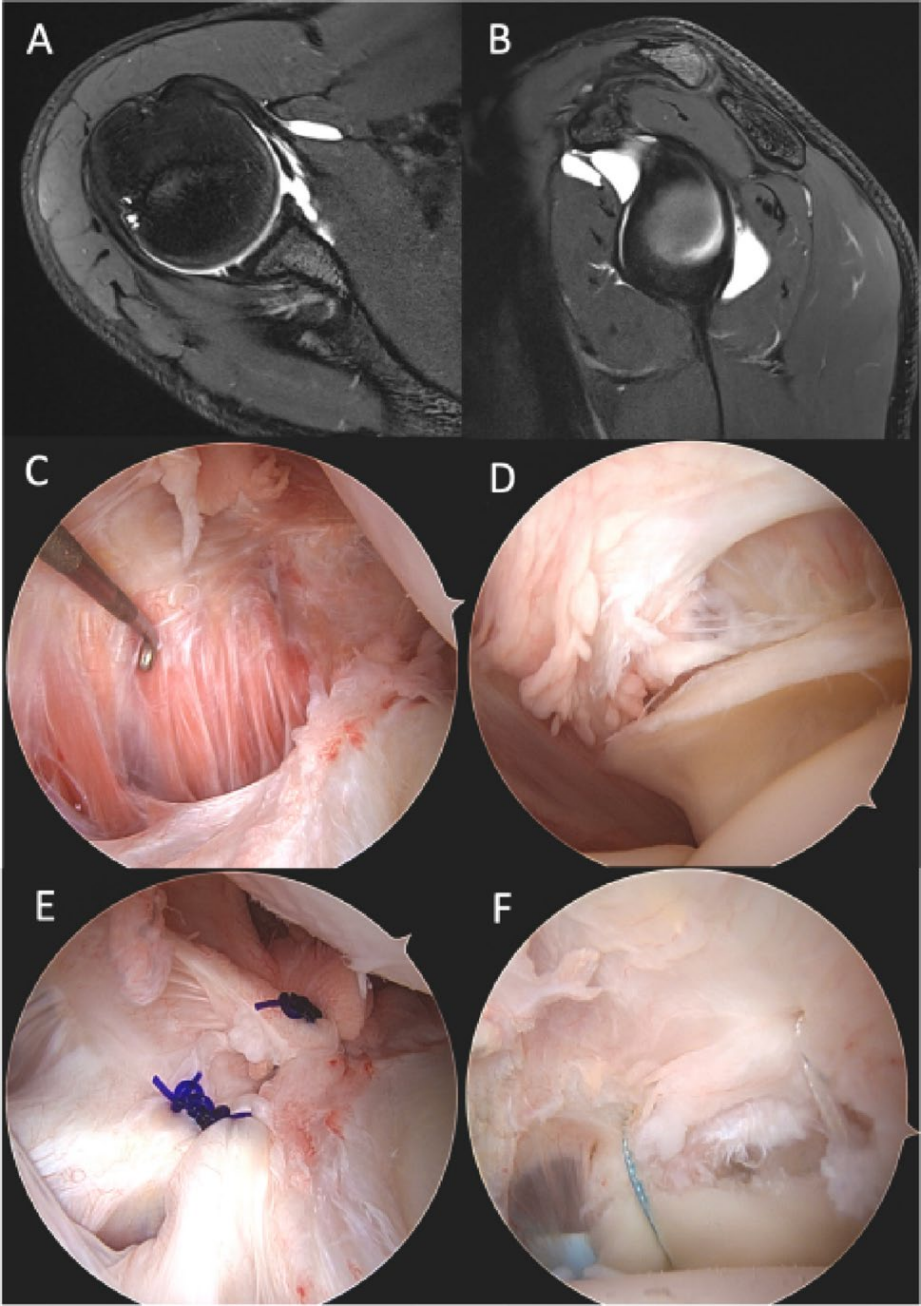
A 14-year-old male patient presented with anterior instability of the right shoulder. Pathologies identified in axial (A) and sagittal (B) MRI scans included an anteroinferior intracapsular rupture (C), and an articular-sided mid-substance partial tear of the supraspinatus tendon (D), all confirmed during the diagnostic round. Surgical management involved side-to-side closure of the capsular rupture (E), and reconstruction of the partial supraspinatus tendon tear (F)

Arthroscopic soft tissue Bankart repair is the most common option for traumatic instability with minimal or no glenoid bone loss [59]. It is typically considered after the first instability episode in patients over 14 years of age with pathological anatomical changes and participation in high-level sports [42; 59]. Moreover, Kraus et al. reported excellent outcomes in patients with an average age of 12 years (age range, 11 to 15 years) who were treated with open or arthroscopic stabilization using suture anchors after primary post-traumatic shoulder instability and showed no additional redislocation events at final follow-up [27]. Furthermore, in cases of suspected concomitant shoulder pathologies (e.g., rotator cuff injuries or biceps tendon pathology), arthroscopy plays a crucial role in addressing these conditions (Fig. 3). However, Cordischi et al. reported in a study of surgical versus nonsurgical management of primary traumatic shoulder dislocations in 13 skeletally immature patients (age range, 10.9–13.1 years) a recurrence rate of 21%, and showed that the cohort managed conservatively better clinical scores compared with the surgical cohort [10]. In conclusion, for primary shoulder instability, arthroscopic stabilization is recommended for patients aged 12–16 years, whereas conservative therapy shows better outcomes for patients less then 12/13 years. The age limit is still poorly understood and needs further investigation.

#### Recurrent

The major risk after the first episode of traumatic instability is recurrence [31]. Although previous literature largely consists of small retrospective series, the overall trend for patients with open physes (e.g. around 14 or less) suggests lower rate of recurrent instability [37]. Deitch et al. conducted a study with 32 adolescent patients and found that 15 patients with an open proximal humeral physis at the time of injury had a recurrence rate of 53%, whereas the 17 patients with closed physes had a recurrence rate of 88%, approaching that of young adults [11]. Recently, Torrance et al. reported in a case series of 67 patients < 18 years of age that after arthroscopic stabilization of a Bankart lesion in adolescent rugby players, recurrent instability in those < 16 years of age was 2.2 times more likely than the older adolescent patients in the cohort [60]. The youngest patient included in the aforementioned study was 14 years of age, which is why the impact of age less than 14 years was not clear. Lampert et al. retrospectively studied 54 pediatric first-time dislocators and found a recurrence rate of 0% among the 12 patients less than 14 years old with open physes [28].

**Figure 4:**
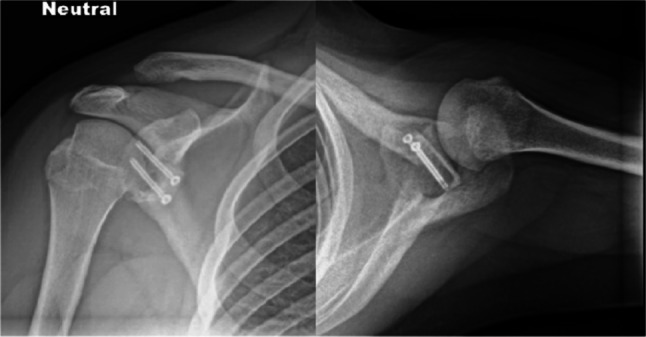
Post-operative radiographs following a Latarjet procedure for a 15-year-old adolescent athlete with recurrent shoulder instability and glenoid bone loss following prior arthroscopic Bankart repair. Figure taken from Kay et al. 2023 [25]. Reprinted with permission from Springer Nature [25]

In the cases of glenoid defects, remplissage or a bone block procedure can be discussed [6]. Adding a remplissage procedure to a Bankart repair is considered when a Hill-Sachs lesion is found to be engaging with subcritical glenoid bone loss [32]. Patients with significant glenoid bone loss or failed prior arthroscopic stabilization are likely to benefit from osseous glenoid reconstruction using coracoid bone transfer, also known as the Latarjet procedure, or iliac crest bone graft transfer (ICBGT) [35] (Fig. 4). Given the increased use of the Latarjet procedure in adolescents in recent years [12, 18, 63], some may question how young can patients be, and whether or not a Latarjet can be done in patients less than 14 years old with open physes, since the coracoid process completely fuses in both sexes at 16–17 years old [9, 47, 67]. However, until recently, there had been no literature evaluating the safety and efficacy of the Latarjet procedure and ICBGT in this pediatric population. The use of allogenic bone material could bring additional benefits in anatomical reconstruction of the glenoid in the setting of a recurrent shoulder instability in paediatric population, eliminating donor site morbidity with possible injury to the growth plates. However, there is no clinical data available demonstrating the use of allogenic bone material for shoulder instability in this population.

### Posterior instability

Posterior instability in patients less than 14 years old is rare and Its treatment is similar to adults. Few studies have focused on shoulder dislocation in these patients, as most studies combine them with adolescents or both adult and pediatric patients from a heterogenous population [36].

Regardless of the cause or nature of the instability, the first-line treatment is conservative for at least six months. This includes modifying sports activities, relearning daily movements to avoid dislocation, undergoing rehabilitation (focusing on proprioception, and strengthening of posterior dynamic stabilizers), and using pharmaceutical pain relief for painful cases. This approach is particularly important because some cases of hypermobility are temporary and may resolve after puberty. Conservative treatment should be maintained as long as possible to allow for the complete maturation of the capsule and ligaments [29].

Among the posterior instability types according to Moroder et al., type B1 is the most encountered functional shoulder instability in the paediatric population. Due to the lack of structural defects, the so-called shoulder pacemaker treatment is recommended, which has been shown to provide a better outcome than conventional physiotherapy alone. Motion-triggered neuromuscular electrical stimulation is applied during exercises to reactivate physiological muscle activation patterns and thus stable shoulders via a feedforward learning effect [44; 49].

Surgical intervention may be needed for ongoing or recurrent instability after failure of well-conducted conservative treatment and in those with well-defined anatomical lesions [13; 30]. Wright et al. reported on a 10-year-old with recurrent instability treated surgically with a glenoid-based posterior capsular shift [65]. A modified McLaughlin procedure might be an option for managing persistent instability in adolescent patients, but it could pose challenges for younger children [50]. The proximity to the growth plate increases the risk of growth-related injuries, potentially leading to limb shortening or angular deformity. As far as we know, there are no reports of the McLaughlin procedure being used in pediatric patients.

### Multidirectional instability

Multidirectional instability (MDI) is observed in patients with generalized ligamentous laxity (either idiopathic or as part of Ehlers-Danlos syndrome or other syndromes) or those with repetitive microtrauma. However, there is limited literature specifically addressing MDI in the pediatric population, with most studies including both adults and adolescents.

Nonsurgical mangement is the first-line treatment of MDI without structural damage and achieves satisfying clinical results. Physical therapy focusing on stabilizing peri-scapular musculature, improving neuromuscular control, proprioception, and activity modification is necessary to optimize the role of the dynamic stabilizers [26].

Surgical stabilization for MDI can be considered, when conservative treatment fails and patients continue to have symptoms of instability and pain that affects daily activities or sports. It typically focuses on reducing capsule volume, most often in its inferior axial portion [29]. Arthroscopic capsular plication is the gold-standard as it confers the advantage of allowing visualisation and plication of the capsule in all shoulder quadrants [53]. However, traditionally, open inferior capsular shift may also be used [46]. Both techniques are associated with good clinical postoperative results, which is mostly described for adolescents [43; 62], whereas no studies can be found for patients less than 14 years old. Surgical stabilization should be followed by physical therapy, which improves symptoms and reduces the risk of recurrence [1, 15].

## Conclusion

Paediatric shoulder instability represents a complex and clinically challenging condition, in which establishing the underlying etiology remains difficult. The etiology, the direction of instability, and associated lesions guide the treatment, which is most often initially conservative. Children aged ≤13 years are less likely to experience recurrent instability than those aged 14–18. Therefore, for primary shoulder instability, arthroscopic stabilization can be used for the patients aged 12–16 years, whereas conservative therapy shows better outcomes for patients aged less then 12–13 years. Patients with significant glenoid bone loss, large Hill-Sachs lesions or failed prior arthroscopic stabilization may benefit from osseous glenoid reconstruction using Latarjet procedure. A detailed discussion of the risks and benefits of nonoperative versus operative management is critical to match the shared decision-making with the patient’s injury pattern, risk factors, and athletic goals.

### Figures

## Data Availability

No datasets were generated or analysed during the current study.
